# Lactylation as a metabolic-epigenetic switch in cancer: dual roles in cell death resistance and therapeutic vulnerability

**DOI:** 10.1038/s41419-026-08494-7

**Published:** 2026-03-06

**Authors:** Chengjiao Yang, Ruici Yang, Binbin Zheng, Hongxiao Jiang, Xianteng Wang, Weiren Huang

**Affiliations:** 1 Department of Urology, Shenzhen Institute of Translational Medicine, Medical Innovation Technology Transformation Center, Shenzhen Second People’s Hospital, The First Affiliated Hospital of Shenzhen University, Institute for Advanced Study, Synthetic Biology Research Center, International Cancer Center of Shenzhen University, Shenzhen, Guangdong Province China; 2 Shenzhen Key Laboratory of Synthetic Genomics, Guangdong Provincial Key Laboratory of Synthetic Genomics, State Key Laboratory of Quantitative Synthetic Biology, Shenzhen Institute of Synthetic Biology, Shenzhen Institutes of Advanced Technology, Chinese Academy of Sciences, Shenzhen, Guangdong Province China; 3 Molecular Cancer Research Center, School of Medicine, Shenzhen Campus of Sun Yat-sen University, Sun Yat-sen University, Shenzhen, Guangdong Province China; 4 Guangdong Key Laboratory of Systems Biology and Synthetic Biology for Urogenital Tumors, Shenzhen, Guangdong Province China; 5 GuangDong Engineering Technology Research Center for clinical application of cancer genome, Shenzhen, Guangdong Province China

**Keywords:** Cancer epidemiology, Cell death

## Abstract

Protein lactylation emerges as a pivotal metabolic rheostat, translating microenvironmental lactate flux into stable programs that orchestrate cancer treatment resistance. This review synthesizes recent advances under the framework of “Lactylation Switch in Cancer Vulnerabilities.” We dissect the dominant enzymatic pathways (AARS1/2, KATs, HDACs) and non-enzymatic mechanisms (MGO/LGSH), alongside their critical structural underpinnings. Furthermore, we delineate how lactylation signals are interpreted by specific readers (e.g., TRIM33), directly reprogram non-histone protein function through structural metamorphosis (e.g., disrupting p53, enhancing XLF), and engage in complex crosstalk with other PTMs, as exemplified by the synergistic interplay between histone H3 lysine 18 lactylation (H3K18la) and histone H3 lysine 27 acetylation (H3K27ac) in T-cell acute lymphoblastic leukemia (T-ALL). This interplay coordinately drives metabolic-epigenetic reprogramming, which specifically rewires intra- and extratumoral survival mechanisms. Lactylation fundamentally establishes a therapy-adaptive state by simultaneously enhancing intrinsic resistance (e.g., BLM K24la-mediated DNA repair) and extrinsic resistance (e.g., histone lactylation-driven PD-L1 upregulation). Critically, preclinical and clinical studies in validated models demonstrate that targeting this lactylation network (e.g., LDHA inhibition with stiripentol, KAT inhibitors, or site-specific blockers) yields striking synergistic effects, potentiating tumor sensitivity to chemotherapy, radiotherapy, and immunotherapy. Looking forward, we outline key translational paths, including deciphering stringent enzyme-substrate specificity for targeted inhibition, developing structure-based drug design, leveraging lactylomic signatures as predictive biomarkers, and addressing current mechanistic and technological gaps. This work not only establishes lactylation as a central mechanism of therapeutic resistance but also provides a novel conceptual paradigm for understanding how metabolic signals dynamically encode cancer cell vulnerabilities, offering transformative opportunities for precision oncology.

**Figure:**
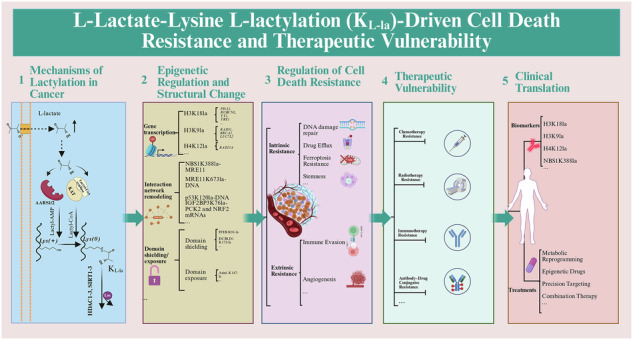
Created in BioRender. Chengjiao, Y. (2026) https://BioRender.com/0lbu6jy.

Created in BioRender. Chengjiao, Y. (2026) https://BioRender.com/0lbu6jy.

## Facts

- Can we exploit the dual functionality of HDACs (lactyltransferases vs. delactylases) to dynamically modulate lactylation in therapy-resistant tumors?
- How do lactylation readers (e.g., TRIM33, Brg1) stereospecifically discriminate Kla from structurally similar PTMs (acetylation, crotonylation) to drive context-dependent transcription?
- Will lactylomic signatures (e.g., BLM K24la, H3K18la) outperform conventional lactate imaging in predicting immunotherapy/chemotherapy response?
- Can site-specific lactylation inhibitors (e.g., irinotecan for BLM K24la) overcome toxicity limitations of broad metabolic/epigenetic modulators?

## Open questions

- Can lactylomic signatures (e.g., BLM K24la, H3K18la) outperform conventional lactate imaging or LDH levels as predictive biomarkers for immunotherapy and chemotherapy response?
- How does the lactylation network dynamically rewire and prioritize different resistance pathways (e.g., DNA repair vs. immune evasion) in response to specific selective pressures within the TME?
- What are the precise molecular determinants that dictate whether a lactylation event on a non-histone protein leads to functional activation (e.g., XLF) or disruption (e.g., p53)?

## Introduction

Post-translational modifications (PTMs) are established master regulators of oncogenesis, with canonical players such as phosphorylation, ubiquitination, and acetylation governing malignant phenotypes [1–3]. However, these modifications primarily respond to signaling cues, leaving a gap in our understanding of how core metabolic fluxes are directly translated into stable pathological states. The discovery of lactate-driven lysine lactylation has unveiled this missing dimension, establishing a direct molecular conduit from metabolism to epigenetic and functional reprogramming [4].

This paradigm shift positions lactate—once dismissed as a waste product [5]—as a central pleiotropic signaling metabolite and the obligate precursor for a novel PTM [6]. Its conversion into lactyl-AMP [7–9] and lactyl-CoA [10, 11] licenses widespread protein modification, spanning histones, DNA repair machinery (e.g., BLM [12]), and transcription factors (e.g., YAP [7]).

We thus conceptualize lactylation as a “metabolic translator” that deciphers lactate abundance into therapeutic resistance. It executes this role through a hierarchical mechanism: sensing the lactate-rich tumor microenvironment (TME) to install site-specific modifications; driving functional rewiring via both histone-mediated transcription and direct alteration of non-histone protein function; and ultimately orchestrating integrated resistance networks across intrinsic (e.g., DNA repair, ferroptosis evasion) and extrinsic (e.g., immunosuppression) axes. Targeting this translator promises to reclaim cancer vulnerability, transforming a metabolic bastion of resistance into a therapeutic Achilles’ heel.

## The lactylation machinery: from molecular establishment to functional integration

The establishment, removal, and interpretation of lysine lactylation are governed by a sophisticated network of enzymatic reactions, metabolite-driven chemical modifications, reader proteins, and structural mechanisms. This machinery collectively translates metabolic flux into profound phenotypic outputs, with key discoveries chronicled in Table 1.

**Table 1:** Evolution of lactylation mechanisms from 2019 to 2025.

Year	Mechanism Development	Notes
2019	Zhang D et al. first reported p300 as a potential writer of histone lactylation (4).	First reported KAT as the potential writer of histone lactylation.
2020	Gaffney DO et al. first reported LGSH-mediated non-enzymatic transfer of lactyl groups to protein Lys residues (generating “LactylLys”) (89).	Mechanistic breakthrough in non-enzymatic lysine lactylation.
2021	Moreno-Yruela C et al. reported HDAC1-3 and SIRT1-3 as lactyl-erasers. (HDAC3 exhibits higher efficiency in removing D/L-lactyllysine in vitro.) (13)	Erasers (HDAC1-3/SIRT1-3).
2023	Sun L et al. first reported that knockdown of AARS1/2 inhibits the lactylation modification of METTL16K229 (49).	AARS1/2 is first reported as lactyltransferasers.
2024	Three independent studies revealed the moonlighting role and specific molecular mechanism of AARS1/2 as lactyltransferases (7–9).	First enzymatic mechanism elucidation of AARS1/2 as lactyltransferases.
	Zhang D et al. reported that K_CE_ is directly induced by MGO (16).	Mechanistic breakthrough in non-enzymatic lysine lactylation.
	Hu X et al. reported that Brg1 acts as a reader for H3K18la (19). Zhai G et al. reported that DPF2 acts as a reader for H3K14la (20). Nunez R et al reported that TRIM33, a bromodomain - containing protein, acts as a novel reader of histone lysine lactylation (21).	Breakthrough in Readers (Brg1/DPF2/TRIM33).
	Sun S et al. reported that HDAC6 of the HDAC family exhibits dual enzymatic activity in vitro, but functions primarily as a lactyltransferase in cells (15).	in vitro studies suggest delactylase activity under low-lactate conditions, but cellular evidence supports a dominant lactyltransferase role in high lactate cells.
2025	Two independent studies identified two genuine lactyl - CoA synthetases (ACSS2/GTPSCS) and their cooperative mechanism with KATs (KAT2A/p300) in lactylation (10, 11).	Identification of lactyl-CoA synthetases (ACSS2/GTPSCS).
	Zhao Q et al. reported that during the inflammatory response, the K_D-la_ generated non - enzymatically by LGSH can be greatly amplified by nearby cysteine residues. Initially, cysteine residues react with SLG to form a reversible S - lactylthiol intermediate, and then the lactyl molecule undergoes SN transfer to the proximal lysine (17).	Mechanistic breakthrough in non-enzymatic lysine lactylation.

### Establishing the modification: enzymatic, non-enzymatic, and eraser pathways

**Fig. 1:**
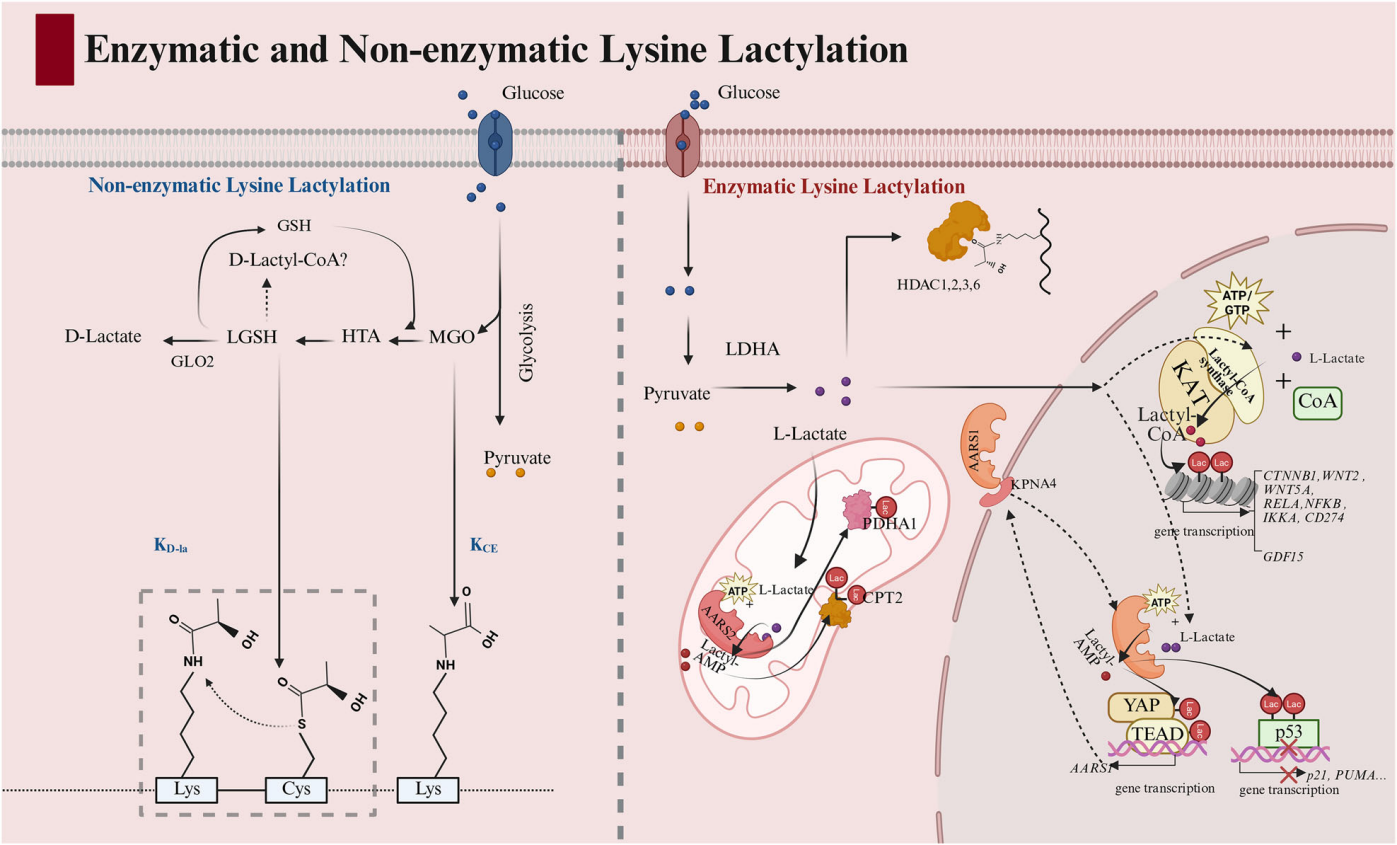
Enzymatic and non-enzymatic lysine lactylation pathways. Lysine lactylation proceeds via two distinct mechanisms: enzymatic (red panel) and non-enzymatic (blue panel). The enzymatic lactylation is primarily mediated by complexes including AARS1/2, KAT-lactyl-CoA synthetase complexes, and HDACs, which utilize glycolysis-derived L-lactate to generate K_L-la_. In contrast, non-enzymatic lactylation involves direct attack on lysine residues by metabolic byproducts MGO and its downstream product LGSH, producing K_CE_ and K_D-la_ modifications. Notably, K_D-la_ production efficiency is enhanced by adjacent cysteine (Cys) residues. Created in BioRender. Chengjiao, Y. (2026) https://BioRender.com/5alwg2k.

Lysine lactylation deposition and removal are regulated by a sophisticated network. The modification is installed through two fundamental routes: enzymatic precision, conferring substrate and context specificity (Fig. 1, red panel), and non-enzymatic chemistry, directly coupling reactive glycolytic metabolites to protein function (Fig. 1, blue panel). Dedicated eraser enzymes complement these pathways by dynamically removing the modification to ensure precise regulation.

#### AARS1/2 as direct lactyltransferases

Enzymatic lactylation is catalyzed by three major classes of writers, each employing distinct strategies to utilize lactate. The lanyl-tRNA synthetases 1and 2 (AARS1/2) enzymes function as ATP-dependent lactyltransferases, directly activating lactate to a lactyl-AMP intermediate before transferring the lactyl group to specific lysine residues [7–9]. This activity is spatially regulated through nuclear import: elevated lactate binding induces a conformational change that exposes a C-terminal nuclear localization signal (NLS; RRIVAVTGAEAQKA) in AARS1, enabling KPNA4 recognition. The AARS1–KPNA4 complex then facilitates nuclear import, allowing lactylation of nuclear substrates like YAP and TEAD to rewire oncogenic transcription [7].

#### KAT-Lactyl-CoA synthetase metabolons for efficient histone lactylation

A separate, highly efficient lactylation pathway involves lysine acetyltransferases (KATs) utilizing lactyl-CoA synthetases—including acetyl-CoA synthetase 2 (ACSS2) and guanosine triphosphate (GTP)-specific SCS (GTPSCS)-mediated route—each employing distinct mechanisms to generate lactyl-CoA (summarized in Table 2) [10, 11].

**Table 2:** Lactyl-CoA synthesis mechanism and influencing factors.

Pathway	Mechanism	Influencing factors
ACSS2 pathway [10]	1. Nuclear Translocation Regulation: EGFR activation triggers ERK-mediated phosphorylation of ACSS2 at S267, promoting importin α5 binding and nuclear translocation. 2. Substrate Binding: ACSS2 binds L-lactate, CoA, and ATP. 3. Intermediate Formation: Lactate carboxyl group reacts with ATP γ-phosphate to form lactyl-AMP intermediate (releasing PPi). 4. CoA Transfer: CoA thiol displaces AMP, generating lactyl-CoA.	Lactate concentration: preferential lactate utilization over acetate/succinate in nuclear compartments. Structural specificity: changes in conformation between open and closed states affect substrate binding. The closed state enhances lactate/CoA binding, while the open state promotes nuclear translocation. Key residues: D358 stabilizes lactate hydroxyl; Y645/R533 anchor lactyl group for catalysis.
GTPSCS pathway [11]	1. Nuclear localization: GTPSCS G1 subunit contains NLS (SUCLG1 residues 192–195), directing nuclear entry. 2. Substrate binding: Binds L-lactate, GTP, and CoA. 3. Intermediate formation: Lactate carboxyl group reacts with GTP β-phosphate to form lactyl-GTP (releasing PPi). 4. CoA transfer: CoA thiol replaces GTP γ-phosphate, yielding lactyl-CoA and GDP (releasing Pi).	Lactate preference: higher *K*_m_ for lactate (15.3 mM) vs. succinate. Lactate concentration: nuclear lactate concentration dictates substrate selection. Key residues: N308 hydrogen-bonds with lactate hydroxyl; G365/V367 stabilize lactate conformation.
LGSH-Mediated [90]	1. GSH cycle: glycolytic intermediate MGO forms LGSH via GSH cycle. 2. Non-enzymatic transfer: LGSH undergoes thiol-disulfide exchange with CoA, generating lactyl-CoA.	GLO2 activity: deficiency drives LGSH accumulation, promoting non-enzymatic S-to-S acyl transfer to CoA. pH dependency: LGSH-CoA transfer is favored under acidic conditions (p*K*_a_ difference between GSH and CoA thiols: 8.4 vs. 9.8).

This pathway ensures efficiency through a multi-tiered mechanism: (i) a feedforward metabolic loop establishes nuclear substrate supply through EGF-dependent recruitment of LDHA to the ACSS2- lysine acetyltransferase 2A (KAT2A) complex [10], enabling direct pyruvate-to-lactate conversion and minimizing cytoplasmic shuttle dependence; (ii) enzyme-enzyme channeling creates a privileged substrate pool via synthetase-KAT metabolons [10, 11], generating a localized lactyl-CoA microenvironment that bypasses global substrate scarcity; (iii) intrinsic catalytic preference for lactyl-CoA amplifies the process, with KAT2A exhibiting superior kinetics (lactyl-CoA: *K*_m_ = 0.4890 μM, *V*_max_ = 2.946 nM·s^−^^¹^; acetyl-CoA: *K*_m_ = 0.8408 μM, *V*_max_ = 1.600 nM·s^−^^¹^) via specific interactions such as the R533-hydrogen bond [10], while GTPSCS is optimized for lactate (*K*_m_ = 15.32 μM) and—given the high nuclear lactate concentration (40–50 μM) versus low succinate (~0.03 mM)—preferentially generates lactyl-CoA over succinyl-CoA (2–6-fold higher) [11]. This coordinated mechanism ensures highly efficient histone lactylation.

#### HDACs as lactate-sensing bifunctional enzymes

The role of HDACs in lysine lactylation presents a fascinating paradigm of context-dependent bifunctionality, with studies reporting seemingly opposing functions as either lactyltransferases (“writers”) or delactylases (“erasers”). This apparent contradiction can be reconciled by considering lactate concentration as a critical determinant of catalytic direction. While HDAC1-3 demonstrate Zn²⁺-dependent delactylase activity in vitro [13], they shift to lactyltransferase function at physiological lactate levels ( > 0.5 mM) [14]. This lactate-directed switching is further evidenced by HDAC6, which acts as a lactyltransferase above 1 mM, targeting α-tubulin K40 to link glycolysis with cytoskeletal remodeling [15].

Given consistent lactate levels of 10-40 mM, HDACs likely function primarily as lactyltransferases in most cancers. Future studies should clarify the context-dependent balance between HDACs’ lactyltransferase and delactylase activities across cancer cells, immune cells, and normal tissues. Another key question is whether HDACs exhibit substrate preference—differentially regulating histone versus non-histone lactylation—which has profound implications for understanding their biological impact and therapeutic targeting.

#### Non-enzymatic lactylation driven by glycolytic byproducts

Operating in parallel, non-enzymatic lysine lactylation provides a direct, chemistry-driven pathway that directly links glycolytic flux to protein modification without enzyme catalysis. This occurs primarily through two highly reactive glycolytic byproducts. The dicarbonyl metabolite methylglyoxal (MGO) can directly modify lysine residues via Michael addition to form N-ε-carboxyethyllysine (K_CE_) [16]. Separately, the glyoxalase pathway intermediate lactoylglutathione (LGSH) facilitates the generation of D-lactyllysine (K_D-la_) through a unique mechanism involving a nucleophilic cysteine residue, which first forms a transient S-lactylated intermediate before the lactyl group is transferred to a proximal lysine via an SN2 reaction [17]. While the functional roles of these non-enzymatic marks in cancer are still emerging, their existence underscores a direct, inescapable link between metabolic activity and the proteomic landscape, presenting a compelling frontier for future research.

#### Dedicated erasers with stereochemical specificity

The removal of lactyl marks is executed by dedicated erasers with distinct stereochemical preferences. HDAC1–3 are Zn²⁺-dependent delactylases that preferentially hydrolyze K_D-la_, exhibiting >3.5-fold higher catalytic efficiency (*k*_cat_/*K*_M_) than for K_L-la_, a selectivity conferred by a key histidine residue (e.g., H134 in HDAC3) that hydrogen-bonds the D-lactyl hydroxyl [13]. In contrast, SIRT1–3 are NAD⁺-dependent delactylases that favor K_L-la_, as structural analyses of SIRT2 reveal optimal positioning of the L-lactyl carbonyl oxygen (~4.1 Å from NAD⁺) versus the misaligned D-isomer (~6.4 Å), which impedes catalysis [18].

### Decoding the signal: readers and the structural grammar of lactylation

The interpretation and functional output of lactylation signals operate through two parallel mechanisms: the recognition of histone lactylation marks by reader proteins to regulate transcription, and the direct structural alteration of non-histone proteins to remodel their functions.

#### Histone lactylation: recognition and transcriptional reprogramming

The molecular recognition of histone lactylation has emerged through the identification of pioneering readers—including Brg1 [19], DPF2 [20], and TRIM33 [21]—that employ distinct structural motifs to translate metabolite-derived modifications into context-specific transcriptional programs, such as promoting cellular reprogramming, driving tumorigenesis, or regulating macrophage polarization (Table 3).

**Table 3:** Pioneer histone lactylation “reader” proteins and their mechanistic functions.

Reader protein	Target modification	Critical binding motif	Structural mechanism	Biological outcome	Ref
BRG1	H3K18la	Bromine domain	N/A	Promotes mesenchymal–epithelial transition (MET) during cellular reprogramming by activating core pluripotency genes (e.g., OCT4, NANOG, SOX2).	[19]
DPF2	H3K14la	Dual PHD zinc-finger domains (PHD1-PHD2)	The lactyl group of H3K14la is anchored within a hydrophobic pocket in PHD1. The N-terminal α-helix of H3 engages PHD2 via electrostatic and hydrogen-bonding interactions (e.g., D346, L342).	Drives tumorigenesis by activating oncogene expression (e.g., SEMA5A, ROCK1), facilitating cell cycle progression, and anti-apoptotic signaling.	[20]
TRIM33	H3K18la	PHD-bromodomain	Specific recognition is mediated by dual hydrogen bonds between the lactyl carbonyl group and residue E981 in the PHD-bromodomain, conferring selectivity over acetylated lysine.	Critically regulates macrophage polarization from pro-inflammatory M1 to reparative M2 states by modulating inflammatory gene transcription.	[21]

Despite these advances, the structural basis of lactylation decoding remains nascent. Although current readers provide initial mechanistic insights—for instance, DPF2 anchors the histone H3 lysine 14 lactylation (H3K14la) lactyl group in a hydrophobic pocket, and TRIM33 uses residue E981 for specific hydrogen-bonding recognition (Table 3)—their substrate-binding architectures largely lack high-resolution validation. Critical unresolved questions include how readers achieve stereochemical discrimination against structurally similar modifications (e.g., acetylation, crotonylation), and how dynamic modification stoichiometry regulates chromatin engagement. Bridging these gaps requires the systematic identification of additional readers coupled with high-resolution structural elucidation via cryo-EM and crystallography.

#### Functional reprogramming of non-histone proteins via structural metamorphosis

Beyond histones, lactylation extensively reprograms non-histone protein function through profound structural metamorphosis, exhibiting a dual capacity to dismantle native architectures and forge pathological complexes. The tumor-suppressor p53 exemplifies its disruptive potential: lactylation at K120/K139 within its DNA-binding domain introduces steric hindrance and negative charge, distorting the DNA-binding interface and disrupting liquid-liquid phase separation, thereby crippling its transcriptional activity [9]. Conversely, lactylation constructs functional assemblies; in XRCC4-like factor (XLF), K288la enhances electrostatic complementarity with Ku80 to accelerate DNA repair complex assembly [22], while NBS1-K388la stabilizes the MRN complex by exposing cryptic binding sites [23]. This transformative power extends to regulating phase separation, as seen with YT521-B homology (YTH) domain-containing 1 (YTHDC1)-K82la, where lactylation drives aberrant nuclear condensate formation to shield oncogenic mRNAs from decay [24].

### Integration with cellular networks: lactylation in the PTMs symphony

Lactylation does not operate in isolation but is fully integrated into the cellular signaling network through extensive crosstalk with other PTMs.

It orchestrates complex dialogs with other PTMs, dynamically rewiring cellular responses. The crosstalk with ubiquitination is bidirectional: lactylation can stabilize oncoproteins like TFEB by shielding them from E3 ligases [25], or it can promote the degradation of others like YTHDC1 by recruiting specific ubiquitin ligases [26]. In the case of acetylation, lactylation exhibits context-dependent synergy or competition. In T-ALL, H3K18la and H3K27ac co-occupy oncogenic promoters, cooperatively opening chromatin to hyperactivate transcription [27]. In stark contrast, on p53, lactylation competes with activating acetylation at the same lysine residues, functionally antagonizing its tumor-suppressor activity [9].

This intricate PTMs symphony positions lactylation as a central metabolic translator. By bidirectionally controlling protein stability and spatiotemporally coordinating acetyl marks, lactylation fine-tunes pathway activity in direct response to nutrient flux, ultimately reprogramming stress adaptation in therapy-resistant malignancies.

## Lactylation and cancer cell death resistance

**Fig. 2:**
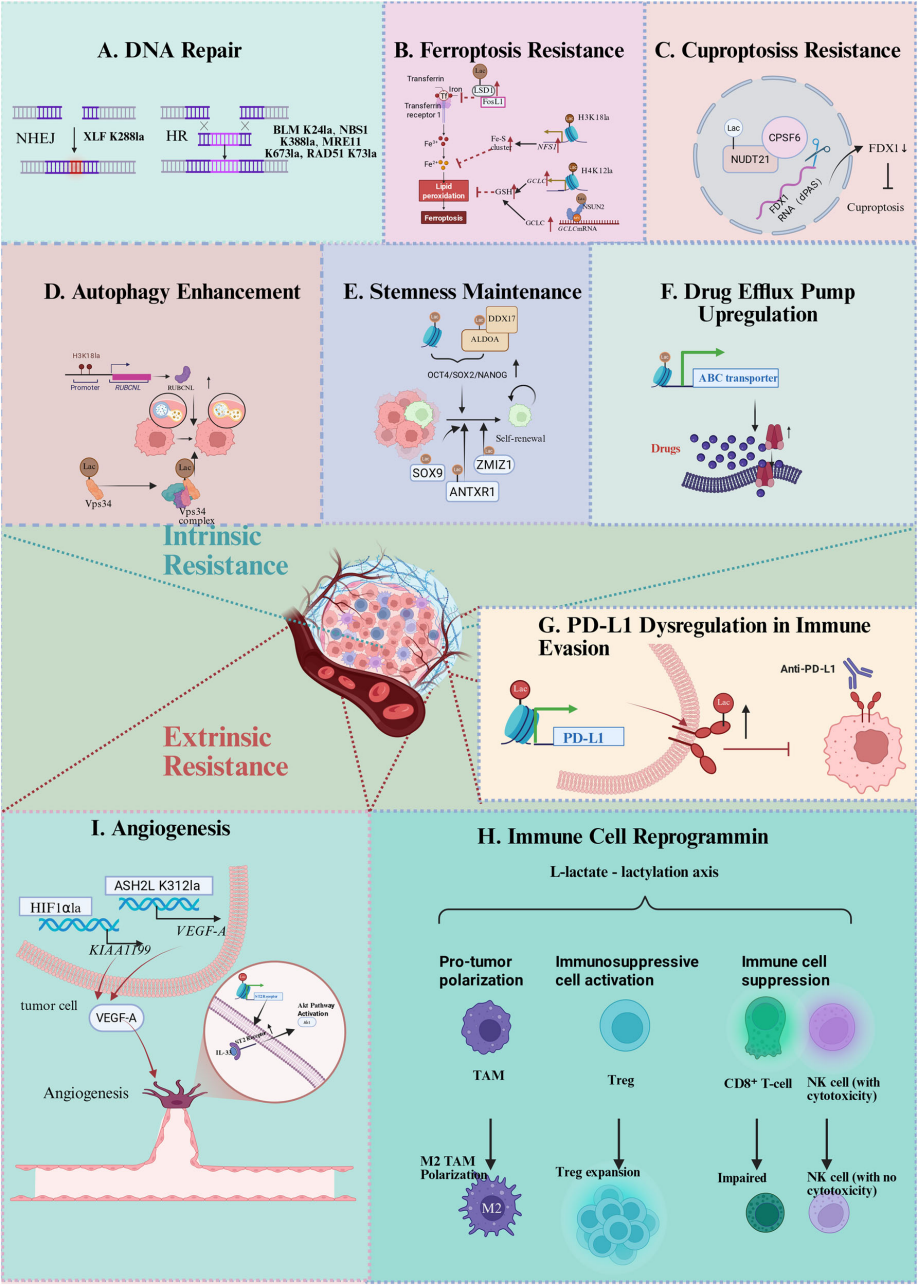
Lactylation-mediated mechanisms of cancer cell death resistance. This schematic illustrates how lactylation drives cancer cell death resistance through diverse mechanisms, encompassing both intrinsic cell survival pathways and extrinsic microenvironmental remodeling. **A** DNA Repair Enhancement. Lactylation augments DNA damage repair pathways to promote survival under genotoxic stress. **B** Ferroptosis Resistance. Lactylation coordinates metabolic rewiring to confer resistance to iron-dependent cell death. **C** Cuproptosis Resistance. Lactylation promotes resistance to copper-induced cell death. **D** Autophagy Enhancement. Lactylation promotes tumor adaptation by activating and sustaining autophagic flux. **E** Stemness Maintenance. Lactylation enforces a cancer stem-like cell state, underpinning therapy evasion. **F** Drug Efflux Upregulation. Lactylation drives the expression of efflux transporters, reducing intracellular drug accumulation. **G** PD-L1 Upregulation. Lactylation elevates PD-L1 levels through transcriptional and post-translational mechanisms, facilitating immune evasion. **H** Immune Cell Reprogramming. Lactylation induces pro-tumor phenotypic and functional shifts in immune cells while suppressing anti-tumor immunity. **I** Pathological Angiogenesis. Lactylation activates pro-angiogenic programs in both tumor and stromal cells to fuel tumor vascularization. Created in BioRender. Chengjiao Y (2026) https://BioRender.com/94mlfod.

Hanahan and Weinberg’s hallmarks of cancer [28] are significantly influenced by protein lactylation, particularly in: (i) sustained proliferative signaling (through cell cycle gene regulation [29]), and (ii) metabolic reprogramming (via glycolytic flux modulation [30, 31]); and (iii) enhancing invasiveness and metastasis by promoting epithelial-mesenchymal transition (EMT) [32–34]. Functioning as a central metabolic-epigenetic hub, lactylation centrally orchestrates tumor cell death resistance. A growing body of evidence (2023–2025) demonstrates its crucial regulatory roles in both intrinsic and extrinsic resistance mechanisms (Fig. 2). This dual functionality underscores lactylation inhibition as a promising strategy to overcome tumor adaptation and circumvent therapeutic challenges.

### Intrinsic resistance: DNA repair and metabolic adaptation

#### DNA repair enhancement

Lactylation orchestrates therapeutic resistance in cancers through epigenetic reprogramming of DNA damage response (DDR) pathways (Fig. 2A).

This PTM exerts dual regulatory effects: modulating histone-dependent transcriptional activation of DDR genes and functionally enhancing non-histone DNA repair proteins. Histone lactylation drives context-specific DDR gene expression across malignancies—exemplified by H3K9la-mediated LUC7L2 induction suppressing mismatch repair in glioblastoma (GBM) [35]; glycolysis-dependent H3K18la accumulation at YY1/YBX1 promoters upregulating anti-apoptotic genes in cisplatin-resistant bladder cancer (BCa) [36]; Histone H4 lysine 12 lactylation (H4K12la) enrichment facilitating MYC recruitment to amplify homologous recombination (HR) capacity in ovarian cancer (OC) [37]; and elevated histone H3 lysine 9 lactylation (H3K9la) directly transactivating core HR genes RAD51/BRCA2 to enhance double-strand break repair efficiency [38]. Concurrently, non-histone lactylation directly potentiates DDR protein activity through multiple mechanisms: enabling nuclear import of repair factors (e.g., XRCC1 K247la promoting radioresistance) [39]; stabilizing core complexes (e.g., NBS1 K388la reinforcing MRE11–RAD50–NBS1 integrity [23]); enhancing enzymatic function (e.g., MRE11 K673la optimizing DNA end resection [40]); and modulating degradation pathways (e.g., BLM K24la reducing ubiquitin-mediated turnover to promote HR repair [12]).

Collectively, lactylation reinforces the structural integrity, functional persistence, and operational efficiency of the DDR machinery across cancer types. However, the hierarchy and potential cooperativity between the epigenetic and functional layers of this lactylation-mediated DDR network remain to be systematically mapped, representing a key gap in understanding its overall topology.

#### Iron metabolism dysregulation and ferroptosis resistance

Lactylation confers ferroptosis resistance through coordinated metabolic rewiring that simultaneously depletes catalytic iron pools and amplifies antioxidant defenses (Fig. 2B). This dual-pathway strategy intercepts the lipid peroxidation cascade at multiple nodes.

First, lactylation restricts bioavailable iron through epigenetic mechanisms. In melanoma, lactylation of lysine-specific demethylase 1 (LSD1) promotes its stabilization and complex formation with FosL1, leading to epigenetic repression of the transferrin receptor 1 (TFRC) and consequently limiting iron influx [41]. In hepatocellular carcinoma (HCC), post-ablative H3K18la transcriptionally activates NFS1 to enhance iron-sulfur cluster biogenesis [42], while in triple-negative breast cancer (TNBC), H3K18la upregulates ZFP64, increasing ferritin heavy chain 1 (FTH1)-mediated Fe²⁺ sequestration [43].

Concurrently, lactylation fortifies cellular antioxidant capacity via glutathione-centric strategies. Tumor cells exploit lactylation to boost GSH synthesis, as evidenced by H4K12la-dependent glutamate-cysteine ligase (GCLC) upregulation in colorectal cancer (CRC) stem cells [44]; NSUN2 K508la-mediated stabilization of GCLC mRNA in gastric cancer (GC) [45]; and MEK/ERK-driven GCLM K34 lactylation strengthening γ-glutamylcysteine ligase activity in KRAS^G12D^-mutant cancers [46]. Beyond GSH production, lactylation activates ROS-quenching systems, as demonstrated in HCC, where PRDX1 K67la promotes NRF2 nuclear translocation, inducing SLC7A11 (for cysteine import), GPX4 (for peroxide clearance), and HMOX1 (for ROS reduction) [47].

Collectively, this metabolic-epigenetic crosstalk establishes a robust defense system: lactylation depletes pro-ferroptotic iron while enhancing redox buffering capacity, enabling tumors to evade iron-dependent cell death across diverse contexts. A critical, unresolved question is how the cell prioritizes these parallel pathways—iron sequestration versus antioxidant synthesis—in response to dynamic metabolic stresses, suggesting the existence of an upstream regulatory switch.

#### Dual roles of lactylation in cuproptosis

The lactate-lactylation axis exhibits functional plasticity in cuproptosis, serving as either a resistance mediator or sensitizer in a context-dependent manner.

In glycolytic tumors like esophageal squamous cell carcinoma (ESCC), lactylation of NUDT21 at K23 stabilizes a 3’-end processing complex that selects distal polyadenylation sites in FDX1 mRNA, leading to transcript destabilization and conferring cuproptosis resistance (Fig. 2C) [48].

Conversely, under copper overload conditions, such as in GC, lactylation is redirected to potentiate cell death. Here, lactylation of METTL16 at K229 enhances its m⁶A-writing activity, which stabilizes FDX1 mRNA and boosts FDX1 protein synthesis, thereby sensitizing cells to cuproptosis [49].

This target-specific duality establishes lactylation as a pivotal determinant of copper vulnerability. While chronic lactate accumulation typically fosters resistance, acute metabolic stress can redirect lactylation toward pro-death signaling, suggesting that targeted manipulation of specific lactylation nodes could convert this adaptive pathway into a therapeutic vulnerability.

#### Autophagy enhancement

Lactylation promotes tumor adaptation by coupling metabolic reprogramming with autophagic signaling through two primary axes: transcriptional activation of autophagy genes and functional modulation of core autophagy machinery (Fig. 2D), establishing stress-responsive survival pathways.

At the transcriptional level, H3K18la in CRC transactivates RUBCNL, promoting BECN1 interaction and class III PI3K recruitment to accelerate autophagosome maturation under hypoxia [50]. Similarly, TFEB K91 lactylation disrupts WWP2-mediated ubiquitination, driving nuclear translocation and upregulation of LC3 and LAMP1 to enhance lysosomal biogenesis [25]. Beyond transcription, lactylation post-translationally regulates autophagy proteins. Nutrient stress triggers a phosphorylation-lactylation cascade: ULK1 phosphorylates LDHA (S196), boosting lactate production and subsequent KAT5/TIP60-mediated VPS34 lactylation (K356/K781), which enhances VPS34 activity to promote autophagosome-lysosome fusion and sustain autophagic flux [51, 52]. This metabolic-autophagy axis is amplified in glycolytic cancers like lung and gastric malignancies, where Warburg-effect-derived lactate fuels therapy adaptation [51, 52]. While lactylation-induced autophagy is typically pro-survival, its potential role in triggering autophagic cell death in specific contexts remains unexplored.

#### Cellular phenotypic plasticity of cancer cell

Lactylation enforces a cancer stem cell (CSC)-like state to drive therapeutic resistance by targeting transcription factors, RNA-binding proteins, and signaling adaptors, thereby activating core stemness programs across malignancies (Fig. 2E).

Liver cancer stem cells (LCSCs) exhibit dual coordination where histone H3 lysine 56 lactylation (H3K56la) establishes chromatin accessibility at pluripotency loci (OCT4/SOX2/NANOG), while ALDOA lactylation induces DDX17 nuclear translocation to synchronize transcriptional and RNA processing programs [53]. This paradigm extends to non-small cell lung cancer (NSCLC) through hypoxia-induced SOX9 lactylation boosting NANOG/CD133 expression [54], and to glioma stem cells, where PTBP1 lactylation reinforces a self-perpetuating “lactate-glycolysis-lactylation” circuit via PFKFB4 upregulation [55]. The functional link to therapy resistance is further demonstrated in CRC, where ANTXR1 lactylation stabilizes the protein and activates a RhoC/ROCK1/SMAD5 axis to sustain stemness and confer oxaliplatin resistance [56], and in breast cancer (BC), where ZMIZ1 lactylation enhances stability and promotes NANOG-driven stemness, leading to tamoxifen resistance [57].

These findings establish the lactylation-stemness axis as a conserved driver of treatment failure. Future studies should determine whether lactylation initiates stemness or amplifies pre-existing stem-like populations—a distinction with critical therapeutic implications.

#### Drug efflux pump upregulation

Emerging evidence indicates that tumor cells can enter a diapause-like state under chemotherapeutic stress, enabling them to evade apoptosis and reduce drug efficacy [58]. In diapause-like cancer cells (DLCCs), a metabolic-epigenetic cascade is initiated by SMC4 silencing: glycolytic activation leads to lactate accumulation, which in turn fuels histone lactylation. This process drives the overexpression of ATP-binding cassette (ABC) transporters, specifically through H4K12la deposition at the ABCC2, ABCC3, and ABCC10 gene loci via chromatin remodeling, ultimately establishing drug efflux-mediated resistance (Fig. 2F) [59]. A notable paradox arises from this mechanism: although SMC4 downregulation inhibits cellular proliferation, it concurrently activates this lactylation-driven resistance axis, presenting a complex adaptive response to therapy.

### Extrinsic resistance: immune evasion and microenvironment remodeling

Extrinsic resistance is orchestrated by lactylation through a cooperative defense program that simultaneously dysregulates tumor cell immunogenicity (e.g., PD-L1 upregulation), reprograms immune cell function, and activates pathological angiogenesis—collectively establishing a therapy-resistant niche.

#### PD-L1 dysregulation in immune evasion

Aberrant PD-L1 overexpression, a hallmark of immune evasion, is regulated by lactylation through two principal mechanisms: epigenetic transcriptional activation and post-translational stabilization of the protein itself (Fig. 2G).

This transcriptional activation is driven by distinct upstream pathways across cancers. In acute myeloid leukemia (AML), STAT5-driven glycolysis facilitates E3BP nuclear translocation and histone H4 lysine 5 lactylation (H4K5la) deposition at the PD-L1 promoter [60]. Solid tumors also frequently utilize histone lactylation, albeit through diverse signaling inputs: GBM employs EGFR-amplified ACSS2-KAT2A complexes to drive lactyl-CoA-dependent H3K18/14la and enhance PD-L1 expression [10]; NSCLC leverages H3K18la to epigenetically activate POM121, which promotes MYC-driven PD-L1 transcription [61]; HCC exploits PRMT3-mediated PDHK1 dimethylation to accelerate lactate production, fueling H3K18la-dependent PD-L1 transactivation that engages PD-1 to suppress T-cell cytotoxicity [62]. In a striking example of post-translational control, CRC cells employ direct PD-L1 lactylation to stabilize the protein, uncoupling its abundance from transcriptional regulation [63]. The fundamental question of what determines the dominant regulatory mode (transcriptional vs. post-translational) in a given tumor type remains open, likely dictated by cell-specific signaling networks and enzymatic machinery.

#### Immune cell reprogramming in the TME

Lactylation remodels the immunosuppressive TME through three interconnected axes: pro-tumor polarization, immunosuppressive cell activation, and direct effector cell suppression (Fig. 2H). These axes form a self-reinforcing circuit wherein tumor-derived lactate drives lactylation on histones and non-histone proteins, reprogramming immune cell phenotypes across cancer types.

In pro-tumor polarization, tumor-derived lactate orchestrates M2 macrophage polarization primarily by inducing H3K18la within macrophages themselves, which drives the transcription of distinct pro-tumorigenic genes across multiple cancer types. In CRC, macrophage H3K18la suppresses RARγ transcription, unleashing the TRAF6–NF-κB–IL-6–STAT3 signaling axis [64]. In HCC, it directly activates M2 phenotype genes such as CD206 and ARG1 [65]. More broadly, in TAMs, H3K18la can upregulate METTL3 to potentiate immunosuppressive JAK–STAT signaling [66], or activate ARG1 expression to foster an immunosuppressive niche, as also observed in cervical cancer (CC) [67]. A distinct indirect pathway operates in pancreatic cancer (PC), where lactate induces H3K18la within tumor cells, activating ACAT2 transcription to promote cholesterol synthesis and sEV-mediated reprogramming of TAMs toward an M2 state [68]. Collectively, these findings position H3K18la—whether acting intrinsically in macrophages or extrinsically via tumor cells—as a central epigenetic regulator that translates metabolic signals into sustained immunosuppressive programming.

In immunosuppressive cell activation, lactate fuels the expansion and functional enhancement of immunosuppressive cells, particularly Tregs and neutrophils, via lactylation-dependent pathways. In GBM, H3K18la-dependent CCR8 upregulation in Tregs disrupts the Th17/Treg balance, facilitating immune evasion [69]. In malignant pleural effusion (MPE), lactylation activates the NF-κB p65/TNFR2 axis, enhancing Treg suppression and CD8 + T-cell inhibition [70]. In HCC, MOESIN K72 lactylation potentiates TGF-β/SMAD3 signaling, driving Treg differentiation and stability [71]. NSCLC exhibits APOC2 K70 lactylation, which triggers FFA release to promote Treg accumulation and anti-PD-1 resistance [72]. In addition, glycolytic CD71+ neutrophils in brain tumors employ lactate-induced histone lactylation to upregulate ARG1, effectively suppressing T-cell function and infiltration [73]. These mechanisms reveal lactylation as a master regulator of immunosuppressive cell pools, with crosstalk between metabolic pathways (e.g., glycolysis, lipid metabolism) and epigenetic modifications amplifying Treg and neutrophil-mediated suppression.

In immune cell suppression, lactylation directly impairs effector immune cells by disrupting critical functions such as cytotoxicity, metabolism, and survival. In head and neck squamous cell carcinoma (HNSCC), H3K9la induces IL-11 expression, activating the JAK2/STAT3 pathway to promote CD8 + T-cell exhaustion and checkpoint upregulation [74]. In KRAS-mutant CRC, H3K18la-induced circATXN7 binds to NF-κB p65, inhibiting its nuclear translocation and exacerbating cytotoxic T lymphocyte (CTL) apoptosis [75]. Similarly, H3K18la-driven B7-H3 overexpression in melanoma and HCC impairs CD8 + T-cell cytotoxicity by suppressing T-cell receptor (TCR) signaling [76]. In AML, ROCK1 K73 lactylation mediates mitochondrial fragmentation, crippling natural killer (NK) cell metabolic fitness and killing capacity [77]. In summary, lactylation functions as a potent immunosuppressive mechanism that directly undermines effector immune cell function, survival, and metabolism across diverse cancer types.

Lactylation emerges as a central orchestrator of immunosuppression, bridging metabolic and epigenetic reprogramming in the TME. Notably, H3K18la recurs as a key modification across diverse immune cells [64–66] and cancers [61, 62, 68], highlighting its broad therapeutic potential. The coordinated mechanisms—polarizing macrophages, activating immunosuppressive cells, and crippling effectors—reveal an integrated lactylation network that tumors exploit for immune evasion, framing it as a critical target for TME reprogramming.

#### Lactylation drives pathological angiogenesis

Lactylation bridges tumor metabolism and epigenetic regulation to drive pathological angiogenesis through coordinated actions in tumor and stromal compartments (Fig. 2I). In tumor cells, lactylated HIF1α in prostate cancer enhances KIAA1199 to promote VEGFA release and vascular mimicry [78], while ASH2L-K312la in HCC recruits the MLL complex to the VEGFA locus to enhance its transcription [79]. Simultaneously, melanoma endothelial cells exhibit H3K18la that upregulates tumorigenicity 2 (ST2), hypersensitizing them to IL-33 and triggering Akt-mediated angiogenesis [80]. Together, these complementary mechanisms establish lactylation as a central upstream switch in tumor vascularization.

Beyond angiogenesis, lactylation couples vascular remodeling with survival mechanisms. VEGFA simultaneously promotes angiogenesis and activates tumor cell VEGFRs in an autocrine PI3K/Akt loop to suppress apoptosis [80]. In PCa, the lactylation–KIAA1199 axis enhances lactate utilization and upregulates SLC7A11 via CaMKII–NRF2, conferring dual resistance to nutrient deprivation and ferroptosis [78]. This coordination extends to immune evasion, as melanoma H3K18la promotes angiogenesis while suppressing high endothelial venule formation to reduce lymphocyte infiltration [80]. Thus, lactylation integrates vascular, metabolic, and immune mechanisms into a unified defense network supporting tumor progression.

### Integrated lactylation network in therapeutic resistance and vulnerability

Lactylation establishes a sophisticated defense network that enables tumors to dynamically evade multiple forms of cell death. Rather than operating in isolation, the mechanisms detailed in Table 4 form an integrated system wherein lactate serves as both a metabolic signal and a substrate for lactylation, creating a central regulatory node that coordinates diverse resistance pathways.

**Table 4:** Summary of lactylation-mediated therapeutic resistance in cancer.

Cancer type	Lactylation target	Resistance type	Regulatory mechanism	Ref
GC	NBS1 K388la	Chemo-/radiotherapy resistance	Long-term DNA-damaging therapy → glycolysis ↑ (LDHA ↑ ) → PDHA1 inactivation → lactate↑ → lactyl-CoA → ATM activation → TIP60 Ser86 phosphorylation → TIP60-NBS1 binding → NBS1 K388la → MRN complex assembly → DNA end resection → HR repair ↑ → therapy resistance	[23]
CRC	MRE11 K673la	Chemo-/radiotherapy resistance	DNA-damaging drugs → glycolysis ↑ (LDHA/LDHB/HK ↑ ) → PDHA1 inactivation → lactate↑ → lactyl-CoA → ATM activation → CBP Ser124 phosphorylation → CBP-MRE11 binding → MRE11 K673la → DNA binding affinity ↑ → HR repair ↑ → therapy resistance	[40]
OC	H3K9la & RAD51 K73la	Platinum drug resistance	Platinum drugs → glycolysis ↑ (HK/LDHA ↑ ) → PDHA1 inactivation → lactate↑ → GCN5 catalyzes H3K9la & RAD51 K73la → RAD51/BRCA2 transcription ↑ → HR repair ↑ → platinum resistance	[38]
OC	H4K12la	Niraparib resistance	Niraparib → glycolysis ↑ (PKM/LDHA ↑ ) → PDHA1 inactivation → lactate↑ → H4K12la → Nira-SE activation → RAD23A transcription ↑ → NER repair ↑ → niraparib resistance	[37]
CRC	XLF K288la	5-FU/cisplatin/radiotherapy Resistance	Glycolysis ↑ → lactate↑ → DSBs → ATM activation → GCN5 phosphorylation → GCN5-XLF binding → XLF K288la → NHEJ repair ↑ → therapy resistance	[22]
BCa	H3K18la	Cisplatin resistance	Glycolysis ↑ (HK2/PFKL ↑ ) → lactate↑ → H3K18la → YY1/YBX1 transcription ↑ → DNA repair/drug efflux/EMT ↑ → cisplatin resistance	[36]
BCa	BLM K24la	Anthracyclines resistance	Glycolysis ↑ → lactate↑ → AARS1-catalyzed BLM K24la → BLM stability ↑ → HR repair ↑ → anthracycline resistance	[12]
HCC	H3K18la	Oxaliplatin resistance	Sublethal heat stress → glycolysis ↑ (HK1/PFKL ↑ ) → lactate↑ → H3K18la → NFS1 transcription ↑ → Fe-S cluster biosynthesis → ferroptosis suppression → oxaliplatin resistance	[42]
Melanoma	LSD1 K503la	BRAFi/MEKi resistance	Glycolysis restoration → lactate accumulation → LSD1 K503la → enhanced FosL1 binding / disrupted TRIM21 binding → LSD1 stabilization → LSD1-FosL1 complex enrichment at TFRC promoter → TFRC transcription ↓ → iron uptake ↓ → ferroptosis inhibition → BRAFi/MEKi resistance.	[41]
TNBC	H3K18la	Doxorubicin resistance	CAFs → lactate secretion → H3K18la → ZFP64 transcription ↑ → GCH1 & FTH1 expression ↑ → ferroptosis suppression → DOX resistance	[43]
GC	NSUN2 K508la	Doxorubicin/RLS3 resistance	Glycolysis ↑ → lactate↑ → NAA10-NSUN2 interaction → NSUN2 K508la → GCLC m⁵C modification → GSH synthesis ↑ → ferroptosis resistance	[45]
CRC	HDAC1 K412la	Ferroptosis-inducing therapies resistance	Lactate↑ → HDAC1 K412la → HDAC1 activity ↑ → H3K27ac ↓ → FTO/ALKBH5 repression → FSP1 m⁶A retention → FSP1 expression ↑ → ferroptosis resistance	[91]
HCC	PRDX1 K67la	Regorafenib resistance	ZNF207 ↑ → lactate↑ → PRDX1 K67la → NRF2 activation → ferroptosis suppression → regorafenib resistance	[47]
CRC	H4K12la	Irinotecan resistance	SMC4 attenuation → lactate↑ → H4K12la → ABC transporters transcription ↑ → drug efflux ↑ → irinotecan resistance	[59]
NSCLC	APOC2 K70la	Anti-PD-1 resistance	Glycolysis ↑ → lactate↑ → APOC2 K70la → LPL activation → FFAs release → Treg recruitment → immunosuppressive TME → ICB resistance	[72]
Pancreatic ductal adenocarcinoma (PDAC)	ENSA K63la	Anti-PD-1 resistance	KRAS mutation → glycolysis ↑ → lactate↑ → ENSA K63la → PP2A inhibition → SRC/STAT3 activation → CCL2 ↑ → TAM reprogramming → ICB resistance	[81]
Lung cancer brain metastasis (BM)	H4K12la	Pemetrexed (PEM) resistance	AKR1B10 ↑ → LDHA ↑ → lactate↑ → H4K12la → CCNB1 transcription ↑ → cell cycle acceleration → PEM resistance	[92]
AML	Histone Lactylation	ATRA resistance	Glycolytic reprogramming → lactate↑ → global lactylation ↑ → METTL3 activation → m⁶A modification ↑ → LSC apoptosis inhibition → ATRA resistance	[93]
CRC	H3K18la	Bevacizumab resistance	Bevacizumab → hypoxia → glycolysis ↑ → lactate↑ → EP300-catalyzed H3K18la → RUBCNL transcription ↑ → autophagy ↑ → bevacizumab resistance	[50]
HCC	IGF2BP3 K76la	Lenvatinib resistance	Glycolysis ↑ → lactate↑ → IGF2BP3 K76la → PCK2/NRF2 m⁶A binding ↑ → serine metabolism reprogramming → antioxidant capacity ↑ → lenvatinib resistance	[94]
BCa	YTHDC1 Lactylation	ADC Resistance (e.g., Enfortumab)	Hyperglycemia → lactate↑ → YTHDC1 lactylation → NECTIN4 expression ↓ → ADC targeting weakened → therapy resistance	[26]
CRC	H3K18la & ANTXR1 K453la	Oxaliplatin resistance	CAF-derived lactate → H3K18la → ANTXR1 transcription ↑ → ANTXR1 K453la → RhoC/ROCK1/SMAD5 axis → stemness ↑ → oxaliplatin resistance	[56]

The defining feature of this network is its context-dependent plasticity. The TME functions as a conductor, directing lactylation to specific molecular targets based on selective pressures. When confronted with DNA-damaging agents, the network prioritizes DNA repair enhancement through modifications such as NBS1 K388la [23], MRE11 K673la [40], and XLF K288la [22]. In the face of potent immune infiltrates, lactylation is instead harnessed to upregulate immunosuppressive pathways via APOC2 K70la [72] or ENSA K63la [81]. Under oxidative stress, the network rapidly reinforces anti-ferroptotic defenses through pathways like the H3K18la-NFS1 axis [42]. This plasticity is perhaps best evidenced by instances of strategic crosstalk, where a single modification—such as H3K18la in cisplatin-stressed BCa [36]—transcends a single pathway to orchestrate a multi-pronged resistance program, simultaneously enhancing both DNA repair and drug efflux.

Paradoxically, this coherent network—dependent on lactate and core lactyltransferases—represents a therapeutic vulnerability. Two strategies emerge: broadly targeting this central axis to disrupt multiple resistance pathways simultaneously, or precisely inhibiting pathologically critical lactylation events to disable specific arms with minimal off-target effects. Thus, dismantling the lactylation network, either upstream or at key downstream nodes, provides a rational strategy to overcome multi-faceted resistance and reclaim cancer vulnerability.

## Clinical translation foundation and current strategies

### The rationale and preclinical evidence for lactylation as a biomarker

The established correlation between elevated intratumoral lactate [6, 82, 83] and lactate dehydrogenase (LDH) [84–88] overexpression with aggressive disease and therapy resistance provides a foundational rationale for investigating lactylation. However, bulk lactate measurements offer only a correlative snapshot. Protein lactylation has emerged as a promising, functionally informed candidate biomarker that directly translates dynamic lactate flux into the pathological rewiring underlying therapeutic failure. Compelling preclinical evidence from diverse cancer types underscores its potential across multiple dimensions: (i) Predictive Value for Therapy Response, as seen in the significant association of high H3K18la with bevacizumab resistance in CRC [50] and elevated H3K9la with platinum resistance in OC [38]; (ii) Prognostic Value for Patient Survival, demonstrated by NBS1 K388la [23] and BLM K24la [12] serving as independent risk factors for shortened overall and recurrence-free survival in GC and BCa, respectively; (iii) Functional Specificity and Mechanistic Insight, where lactylation directly impairs the activity of core DNA repair proteins (e.g., NBS1 K388la [23], BLM K24la [12]) to enhance repair proficiency, or rewires transcriptional programs to promote pro-survival pathways such as autophagy (e.g., H3K18la-mediated upregulation of RUBCNL [50]); and (iv) Relevance in the TME, illustrated by lactylation-mediated dysfunction in NK cells from AML patients [77]. This robust, multi-dimensional preclinical evidence establishes a compelling foundation for the clinical translation of lactylation signatures. It not only directly underpins the development of therapeutic strategies but also highlights the critical need to address associated translational challenges.

### Current pharmacological strategies targeting lactylation

**Fig. 3:**
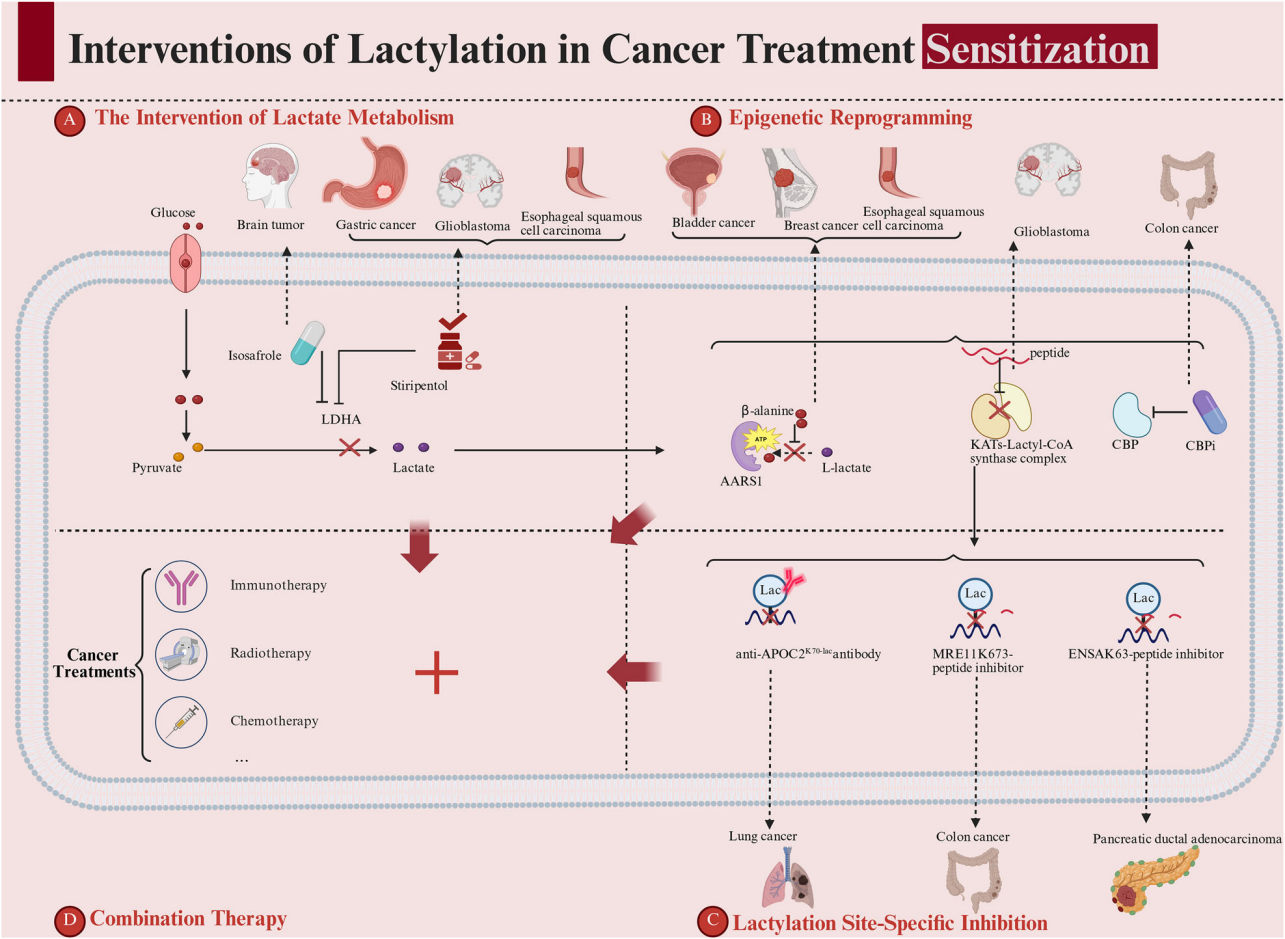
The effects of lactylation interventions on improving cancer treatment sensitivity. This figure visually categorizes four strategic approaches to target lactylation for enhancing cancer treatment sensitivity, aligned with the pharmacological strategies detailed in section “Current pharmacological strategies targeting lactylation”: **A** Metabolic Intervention (Targeting Lactate Production): Illustrates targeting LDHA (e.g., with Stiripentol) to reduce global lactate pools, as seen in cancers like GC. **B** Epigenetic Reprogramming (Targeting Lactylation Machinery): Depicts inhibiting lactyltransferases/metabolons (e.g., AARS1, KATs-Lactyl-CoA synthase complex, CBPi) in cancers such as BCa, BC, and ESCC. **C** Lactylation Site-Specific Inhibition: Shows precise targeting via agents like anti-APOC2 K70la antibody, MRE11 K673-peptide inhibitor, and ENSA K63-peptide inhibitor in lung cancer, CRC, and PDAC. **D** Combination Therapy: Highlights the synergistic potential of combining lactylation-targeted strategies with immunotherapy, radiotherapy, chemotherapy, and other treatments to overcome resistance. Created in BioRender. Chengjiao, Y. (2026) https://BioRender.com/de04hlt.

The profound role of lactylation in therapy resistance has motivated the development of diverse pharmacological interventions, which can be categorized by their point of intervention along the lactylation axis, as visually summarized in Fig. 3.

#### Metabolic intervention: targeting lactate production

The most direct strategy is to reduce the global lactate pool, corresponding to the ‘Intervention of Lactate Metabolism’ axis in Fig. 3A. This is exemplified by the drug repurposing of Stiripentol, an FDA-approved LDHA inhibitor that depletes lactate and reverses chemoresistance in GC [23] and GBM [35] models, effectively synergizing with standard therapies as detailed in Table 5 (LDHA/B row).

**Table 5:** Lactylation-aware combinatorial therapy for clinical translation.

Target/agent	Mechanism of action	Therapeutic impact & synergy	Key translational advantages
**LDHA/B** Stiripentol	Inhibits lactate production, depleting the global lactate pool and reducing lactylation (e.g., NBS1 K388la [23], H3K9la [35]). BBB-permeable [35].	GC: synergizes with cisplatin in resistant PDOs (Bliss synergy) and PDX models [23]. GBM: combined with TMZ in orthotopic models, showing enhanced anti-tumor effects, apoptosis, and DNA damage response [35].	FDA-approved (epilepsy)/BBB-penetrant/Established safety profile; No added toxicity in combos (GBM/GC models); Enables accelerated repurposing for oncology trials.
**AARS1** β-alanine [9, 26]	Competes with lactate for the AARS1 binding pocket, inhibiting its lactyltransferase activity.	Synergizes with doxorubicin in GC models [9] and enhances sensitivity to enfortumab vedotin in BCa models [26].	N/A
**ACSS2-KAT2A** Fusion Peptide [10]	Competitively disrupts the ACSS2-KAT2A interaction, inhibiting histone lactylation (H3K14la/H3K18la) and downstream oncogenic pathways (e.g., PD-L1). Nuclear-targeted.	GBM: combined with anti-PD-1, showing improved tumor control and enhanced immune infiltration.	High specificity: targets only ACSS2-KAT2A interface (spares other functions); Enhanced nuclear delivery by liposomes ( ↓ off-target); Synergy with anti-PD-1 to overcome immune evasion.
**MRE11 K673la** K673-peptide-3 [40]	CPP-fused blocking peptide impairs MRE11 K673la-mediated DNA end resection, compromising homologous recombination repair.	Synergizes with cisplatin/olaparib: increases chemo-sensitivity and DNA damage (γ-H2AX), reduces proliferation and tumor volume in PDX models.	High specificity: Only blocks MRE11-K673la (spares other PTMs/acetylation); Safety: No renal/hepatic toxicity in mice; survival unaffected; Overcomes chemoresistance: Enhances olaparib efficacy in PDX.
**XLF K288la** pep4 (XLF-K288 peptide) [22]	Dual-function peptide: inhibits XLF K288 lactylation and blocks XLF-Ku80 interaction, impairing NHEJ repair.	Synergizes with 5-FU, enhancing anti-tumor effects in CRC models.	High specificity: No off-target effects in K288R-mutant cells; Favorable safety: No hepatorenal toxicity in PDX; Precision targeting: selective for tumors with high K288la.
**APOC2 K70la** Anti-APOC2K70la mAb [72]	Neutralizes lactylated APOC2, blocking its interaction with LPL, suppressing FFA release, and inhibiting Treg expansion.	Synergizes with anti-PD-1, improving tumor control and immune cell profiles in resistant models.	Specificity: only targets K70la modification (no cross-reactivity); Overcoming drug resistance: Remains effective in PD-1-resistant models.
**ENSA K63la** K63-peptide [81]	CPP-fused blocking peptide disrupts ENSA-K63la-PP2A interaction, inhibiting SRC-STAT3 signaling and CCL2-driven TAM infiltration.	Synergizes with anti-PD-1 in PDAC, reducing tumor growth and modulating immune microenvironment.	High specificity: only blocks ENSA-K63la (spares other PTMs); Overcomes PD-1 resistance: Effective in refractory models.
**BLM K24la** Irinotecan [12].	Binds BLM to inhibit K24 lactylation, promoting its MIB1-mediated ubiquitination and degradation, thereby impairing HR repair.	Reverses anthracycline resistance in bladder/breast cancer CDX/PDX models without added hepatotoxicity.	Clinical validation (Phase I): 6 recurrent BCa patients (anthracycline-resistant); Irinotecan liposome (56.5 mg/m² IV biweekly) + intravesical EPI; Result: 100% RFR at 6 months; tumor regression on MRI; only Grade 1-2 AEs.

*AEs* Adverse Events, *BBB* Blood-Brain Barrier, *CPP* Cell-Penetrating Peptide, *CDX* Cell-Derived Xenograft, *PDOs* Patient-Derived Organoids.

#### Epigenetic reprogramming: targeting the lactylation machinery

Strategies that epigenetically target the lactylation writers offer a more precise alternative, as depicted in Fig. 3B. These include (i) inhibiting lactyltransferases—such as AARS1 [9, 26] and KATs (e.g., GCN5 [40])—and (ii) disrupting the metabolon channel that delivers lactyl-CoA to KATs [10, 11]. Agents in this category, including β-alanine [9, 26] and ACSS2-KAT2A fusion peptides [10], have demonstrated robust single-agent and combination efficacy in preclinical models, effectively sensitizing tumors to chemotherapy and immunotherapy (Table 5, AARS1, ACSS2-KAT2A rows).

#### Site-specific inhibition: precision targeting of oncogenic hubs

The most precise approach involves agents that specifically disrupt individual lactylation modifications. This class includes Blocking Peptides (e.g., K288-pe for XLF [22]), Function-Blocking Antibodies (e.g., Anti-APOC2K70la mAb [72]), and Small Molecule Binders (e.g., Irinotecan for BLM K24la [12]). These agents achieve high specificity by targeting unique lactylation sites, thereby impairing oncogenic functions like DNA repair or immunosuppressive signaling, and have shown compelling synergy with standard-of-care treatments across multiple cancer types, as validated in patient-derived xenograft (PDX) models and early-phase clinical trials (Table 5, MRE11, XLF, APOC2, BLM rows).

#### Summary and combinatorial potential

The strategies outlined above, from metabolic modulation to precision targeting, provide a versatile toolkit for overcoming lactylation-driven resistance. As comprehensively summarized in Table 5, a key translational advantage is their favorable preclinical safety profile and demonstrated ability to induce robust synergistic anti-tumor effects when combined with conventional therapies. The powerful synergy of these distinct approaches, culminating in combination therapy, is conceptually captured in Fig. 3D, underscoring the significant clinical potential of integrating lactylation modulation into combinatorial regimens.

## Clinical translation and future horizons

The therapeutic potential of targeting lactylation is substantial, yet its clinical translation requires systematically bridging fundamental biology with clinical application. This process can be conceptualized as a pipeline progressing from target discovery to clinical-trial-ready strategies.

### Foundational science: decoding the lactylation network

A deep, systems-level understanding of lactylation machinery and dynamics forms the bedrock of clinical translation, with several critical gaps requiring resolution.

#### Elucidating reader–writer specificity and PTM crosstalk

The molecular logic of lactylation requires further elucidation. Paramount goals include defining the precise mechanisms underlying writer-substrate specificity and reader discrimination between lactylation and structurally similar PTMs, identifying novel lactyltransferases, lactyl-CoA synthetases, and readers, and mapping functional crosstalk networks with other PTMs (e.g., ubiquitination, acetylation) within therapy resistance pathways.

#### Resolving dynamic lactylation in therapy adaptation

Moving beyond static biomarker measurements is crucial. Future research must capture the lactylome’s dynamic regulation under therapeutic pressure by correlating real-time metabolic imaging (e.g., hyperpolarized [1-¹³C]pyruvate MRI) of lactate flux with longitudinal lactylome profiling from liquid biopsies. This integrated approach will reveal how treatments sculpt the lactylation landscape to foster survival, identifying novel targetable vulnerabilities that emerge under stress.

### Therapeutic development: expanding the druggable toolkit

Translating mechanistic insights into therapeutics requires overcoming significant pharmacological challenges.

#### Rational drug design for an immature target landscape

The pharmacologic targeting of lactylation nodes remains nascent. Key obstacles include disrupting the conserved substrate-binding pockets of writer enzymes and lactyl-CoA synthetases, and addressing the notable scarcity of reader protein inhibitors. A concerted structure-based drug design effort leveraging high-resolution structural biology offers the most direct path to generating first-in-class, highly specific inhibitors.

#### Achieving therapeutic specificity and safety

The bifunctionality of enzymes like HDACs and lactate’s metabolic centrality pose unique safety challenges. Broad metabolic inhibitors (e.g., LDHA antagonists) risk on-target toxicity, while epigenetic inhibitors targeting promiscuous writers like KATs may have off-target effects due to inherent substrate promiscuity. Site-specific inhibitors (e.g., peptides blocking modifications like MRE11 K673la [40]) provide promising precision strategies to minimize systemic disruption.

### Clinical integration: validating biomarkers and trial strategies

Integrating these advances into clinical workflow is essential for demonstrating patient benefit.

#### Clinical qualification of lactylation biomarkers

The promising detection of marks like BLM K24la [12] in early trials must advance through definitive clinical qualification. This requires standardizing robust assays (e.g., clinical-grade IHC or MS), validating their prognostic/predictive value in large retrospective cohorts, and integrating them into prospective biomarker-stratified trials.

#### Designing lactylation-aware clinical trials

Future trials should strategically combine lactylation-modulating agents (e.g., LDHA inhibitors, KATi) with standard therapies while embedding biomarker assessments (e.g., lactylomic signatures, lactate imaging) to identify responsive patient subsets and validate the mechanism of action.

By systematically navigating this pipeline from foundational discovery through therapeutic development to clinical integration, the field can dismantle the lactylation-driven resistome and deliver metabolic-epigenetic precision oncology.

## Conclusion

Lactylation represents a central metabolic-epigenetic switch in cancer therapy resistance, intrinsically enhancing DNA repair, ferroptosis evasion, and autophagy while extrinsically promoting immune evasion and angiogenesis. Its translational promise lies in targeting the network’s core components—lactate, core writers, and pathologically critical modification sites—with pharmacological strategies showing potent preclinical synergy. Future success requires precision approaches: decoding context-specific lactylation events and integrating multi-modal biomarkers into targeted trials to transform this resistance mechanism into a therapeutic vulnerability.

## Data Availability

Data sharing is not applicable in this article as no new data was created or analyzed in this study.

## References

[1] Ge X, Zhang K, Zhu J, Chen Y, Wang Z, Wang P, et al. Targeting protein modification: a new direction for immunotherapy of pancreatic cancer. Int J Biol Sci. 2025;21:63–74. 39744438 10.7150/ijbs.101861 PMC11667816

[2] Hsu JM, Li CW, Lai YJ, Hung MC. Posttranslational modifications of PD-L1 and their applications in cancer therapy. Cancer Res. 2018;78:6349–53. 30442814 10.1158/0008-5472.CAN-18-1892 PMC6242346

[3] Hu Q, Shi Y, Wang H, Bing L, Xu Z. Post-translational modifications of immune checkpoints: unlocking new potentials in cancer immunotherapy. Exp Hematol Oncol. 2025;14:37. 40087690 10.1186/s40164-025-00627-6 PMC11907956

[4] Zhang D, Tang Z, Huang H, Zhou G, Cui C, Weng Y, et al. Metabolic regulation of gene expression by histone lactylation. Nature. 2019;574:575–80. 31645732 10.1038/s41586-019-1678-1 PMC6818755

[5] Brooks GA. The science and translation of lactate shuttle theory. Cell Metab. 2018;27:757–85. 29617642 10.1016/j.cmet.2018.03.008

[6] Chen J, Huang Z, Chen Y, Tian H, Chai P, Shen Y, et al. Lactate and lactylation in cancer. Signal Transduct Target Ther. 2025;10:38. 39934144 10.1038/s41392-024-02082-x PMC11814237

[7] Ju J, Zhang H, Lin M, Yan Z, An L, Cao Z, et al. The alanyl-tRNA synthetase AARS1 moonlights as a lactyltransferase to promote YAP signaling in gastric cancer. J Clin Invest. 2024;134:e174587. 38512451 10.1172/JCI174587 PMC11093599

[8] Li H, Liu C, Li R, Zhou L, Ran Y, Yang Q, et al. AARS1 and AARS2 sense L-lactate to regulate cGAS as global lysine lactyltransferases. Nature. 2024;634:1229–37. 39322678 10.1038/s41586-024-07992-y

[9] Zong Z, Xie F, Wang S, Wu X, Zhang Z, Yang B, et al. Alanyl-tRNA synthetase, AARS1, is a lactate sensor and lactyltransferase that lactylates p53 and contributes to tumorigenesis. Cell. 2024;187:2375–92.e33. 38653238 10.1016/j.cell.2024.04.002

[10] Zhu R, Ye X, Lu X, Xiao L, Yuan M, Zhao H, et al. ACSS2 acts as a lactyl-CoA synthetase and couples KAT2A to function as a lactyltransferase for histone lactylation and tumor immune evasion. Cell Metab. 2025;37:361–76.e7. 39561764 10.1016/j.cmet.2024.10.015

[11] Liu R, Ren X, Park YE, Feng H, Sheng X, Song X, et al. Nuclear GTPSCS functions as a lactyl-CoA synthetase to promote histone lactylation and gliomagenesis. Cell Metab. 2025;37:377–94.e9. 39642882 10.1016/j.cmet.2024.11.005 PMC11798710

[12] Li X, Zhang C, Mei Y, Zhong W, Fan W, Liu L, et al. Irinotecan alleviates chemoresistance to anthracyclines through the inhibition of AARS1-mediated BLM lactylation and homologous recombination repair. Signal Transduct Target Ther. 2025;10:214. 40634292 10.1038/s41392-025-02302-y PMC12241633

[13] Moreno-Yruela C, Zhang D, Wei W, Baek M, Liu W, Gao J, et al. Class I histone deacetylases (HDAC1-3) are histone lysine delactylases. Sci Adv. 2022;8:eabi6696. 35044827 10.1126/sciadv.abi6696 PMC8769552

[14] Gonzatti MB, Hintzen JCJ, Sharma I, Najar MA, Tsusaka T, Marcinkiewicz MM, et al. Class I histone deacetylases catalyze lysine lactylation. J Biol Chem. 2025;301:110602. 40835008 10.1016/j.jbc.2025.110602 PMC12624779

[15] Sun S, Xu Z, He L, Shen Y, Yan Y, Lv X, et al. Metabolic regulation of cytoskeleton functions by HDAC6-catalyzed alpha-tubulin lactylation. Nat Commun. 2024;15:8377. 39333081 10.1038/s41467-024-52729-0 PMC11437170

[16] Zhang D, Gao J, Zhu Z, Mao Q, Xu Z, Singh PK, et al. Lysine L-lactylation is the dominant lactylation isomer induced by glycolysis. Nat Chem Biol. 2025;21:91–9. 39030363 10.1038/s41589-024-01680-8 PMC11666458

[17] Zhao Q, Wang Q, Yao Q, Yang Z, Li W, Cheng X, et al. Nonenzymatic lysine D-lactylation induced by glyoxalase II substrate SLG dampens inflammatory immune responses. Cell Res. 2025;35:97–116. 39757301 10.1038/s41422-024-01060-w PMC11770101

[18] Jennings EQ, Ray JD, Zerio CJ, Trujillo MN, McDonald DM, Chapman E, et al. Sirtuin 2 regulates protein lactoyllys modifications. Chembiochem. 2021;22:2102–6. 33725370 10.1002/cbic.202000883 PMC8205944

[19] Hu X, Huang X, Yang Y, Sun Y, Zhao Y, Zhang Z, et al. Dux activates metabolism-lactylation-MET network during early iPSC reprogramming with Brg1 as the histone lactylation reader. Nucleic Acids Res. 2024;52:5529–48. 38512058 10.1093/nar/gkae183 PMC11162783

[20] Zhai G, Niu Z, Jiang Z, Zhao F, Wang S, Chen C, et al. DPF2 reads histone lactylation to drive transcription and tumorigenesis. Proc Natl Acad Sci USA. 2024;121:e2421496121. 39636855 10.1073/pnas.2421496121 PMC11648877

[21] Nunez R, Sidlowski PFW, Steen EA, Wynia-Smith SL, Sprague DJ, Keyes RF, et al. The TRIM33 bromodomain recognizes histone lysine lactylation. ACS Chem Biol. 2024;19:2418–28. 39556662 10.1021/acschembio.4c00248 PMC11706526

[22] Jin M, Huang B, Yang X, Wang S, Wu J, He Y, et al. Lactylation of XLF promotes non-homologous end-joining repair and chemoresistance in cancer. Mol Cell. 2025;85:2654–72.e7. 40680721 10.1016/j.molcel.2025.06.019

[23] Chen H, Li Y, Li H, Chen X, Fu H, Mao D, et al. NBS1 lactylation is required for efficient DNA repair and chemotherapy resistance. Nature. 2024;631:663–9. 38961290 10.1038/s41586-024-07620-9 PMC11254748

[24] Dai C, Tang Y, Yang H, Zheng J. YTHDC1 lactylation regulates its phase separation to enhance target mRNA stability and promote RCC progression. Mol Cell. 2025;85:2733–48.e7. 40680722 10.1016/j.molcel.2025.06.017

[25] Huang Y, Luo G, Peng K, Song Y, Wang Y, Zhang H, et al. Lactylation stabilizes TFEB to elevate autophagy and lysosomal activity. J Cell Biol. 2024;223:e202308099. 39196068 10.1083/jcb.202308099 PMC11354204

[26] Xing Z, Yang T, Li X, Xu H, Hong Y, Shao S, et al. High-glucose-associated YTHDC1 lactylation reduces the sensitivity of bladder cancer to enfortumab vedotin therapy. Cell Rep. 2025;44:115545. 40215164 10.1016/j.celrep.2025.115545

[27] Wu W, Zhang J, Sun H, Wu X, Wang H, Cui B, et al. Glycolysis induces abnormal transcription through histone lactylation in T-cell acute lymphoblastic leukemia. Genomics Proteom Bioinforma. 2025;23:qzaf029. 10.1093/gpbjnl/qzaf029 PMC12402983 40193528

[28] Hanahan D, Weinberg RA. Hallmarks of cancer: the next generation. Cell. 2011;144:646–74. 21376230 10.1016/j.cell.2011.02.013

[29] Jin J, Bai L, Wang D, Ding W, Cao Z, Yan P, et al. SIRT3-dependent delactylation of cyclin E2 prevents hepatocellular carcinoma growth. EMBO Rep. 2023;24:e56052. 36896611 10.15252/embr.202256052 PMC10157311

[30] Li F, Si W, Xia L, Yin D, Wei T, Tao M, et al. Positive feedback regulation between glycolysis and histone lactylation drives oncogenesis in pancreatic ductal adenocarcinoma. Mol Cancer. 2024;23:90. 38711083 10.1186/s12943-024-02008-9 PMC11071201

[31] Wei S, Zhang J, Zhao R, Shi R, An L, Yu Z, et al. Histone lactylation promotes malignant progression by facilitating USP39 expression to target PI3K/AKT/HIF-1α signal pathway in endometrial carcinoma. Cell Death Discov. 2024;10:121. 38459014 10.1038/s41420-024-01898-4 PMC10923933

[32] Zhao R, Yi Y, Liu H, Xu J, Chen S, Wu D, et al. RHOF promotes Snail1 lactylation by enhancing PKM2-mediated glycolysis to induce pancreatic cancer cell endothelial-mesenchymal transition. Cancer Metab. 2024;12:32. 39462429 10.1186/s40170-024-00362-2 PMC11515152

[33] Huimin W, Xin W, Shan Y, Junwang Z, Jing W, Yuan W, et al. Lactate promotes the epithelial-mesenchymal transition of liver cancer cells via TWIST1 lactylation. Exp Cell Res. 2025;447:114474. 39993459 10.1016/j.yexcr.2025.114474

[34] Wu Z, Peng Y, Chen W, Xia F, Song T, Ke Q. Lactylation-driven transcriptional activation of FBXO33 promotes gallbladder cancer metastasis by regulating p53 polyubiquitination. Cell Death Dis. 2025;16:144. 40021626 10.1038/s41419-025-07372-y PMC11871038

[35] Yue Q, Wang Z, Shen Y, Lan Y, Zhong X, Luo X, et al. Histone H3K9 lactylation confers temozolomide resistance in glioblastoma via LUC7L2-mediated MLH1 intron retention. Adv Sci. 2024;11:e2309290. 10.1002/advs.202309290 PMC11109612 38477507

[36] Li F, Zhang H, Huang Y, Li D, Zheng Z, Xie K, et al. Single-cell transcriptome analysis reveals the association between histone lactylation and cisplatin resistance in bladder cancer. Drug Resist Updat. 2024;73:101059. 38295753 10.1016/j.drup.2024.101059

[37] Lu B, Chen S, Guan X, Chen X, Du Y, Yuan J, et al. Lactate accumulation induces H4K12la to activate super-enhancer-driven RAD23A expression and promote niraparib resistance in ovarian cancer. Mol Cancer. 2025;24:83. 40102876 10.1186/s12943-025-02295-w PMC11921584

[38] Sun C, Li X, Teng Q, Liu X, Song L, Schioth HB, et al. Targeting platinum-resistant ovarian cancer by disrupting histone and RAD51 lactylation. Theranostics. 2025;15:3055–75. 40083924 10.7150/thno.104858 PMC11898288

[39] Li G, Wang D, Zhai Y, Pan C, Zhang J, Wang C, et al. Glycometabolic reprogramming-induced XRCC1 lactylation confers therapeutic resistance in ALDH1A3-overexpressing glioblastoma. Cell Metab. 2024;36:1696–710.e10. 39111285 10.1016/j.cmet.2024.07.011

[40] Chen Y, Wu J, Zhai L, Zhang T, Yin H, Gao H, et al. Metabolic regulation of homologous recombination repair by MRE11 lactylation. Cell. 2024;187:294–311 e21. 38128537 10.1016/j.cell.2023.11.022 PMC11725302

[41] Li A, Gong Z, Long Y, Li Y, Liu C, Lu X, et al. Lactylation of LSD1 is an acquired epigenetic vulnerability of BRAFi/MEKi-resistant melanoma. Dev Cell. 2025;60:1974–90.e11. 40132584 10.1016/j.devcel.2025.02.016

[42] Huang J, Xie H, Li J, Huang X, Cai Y, Yang R, et al. Histone lactylation drives liver cancer metastasis by facilitating NSF1-mediated ferroptosis resistance after microwave ablation. Redox Biol. 2025;81:103553. 39970777 10.1016/j.redox.2025.103553 PMC11876915

[43] Zhang K, Guo L, Li X, Hu Y, Luo N. Cancer-associated fibroblasts promote doxorubicin resistance in triple-negative breast cancer through enhancing ZFP64 histone lactylation to regulate ferroptosis. J Transl Med. 2025;23:247. 40022222 10.1186/s12967-025-06246-3 PMC11871786

[44] Deng J, Li Y, Yin L, Liu S, Li Y, Liao W, et al. Histone lactylation enhances GCLC expression and thus promotes chemoresistance of colorectal cancer stem cells through inhibiting ferroptosis. Cell Death Dis. 2025;16:193. 40113760 10.1038/s41419-025-07498-z PMC11926133

[45] Niu K, Chen Z, Li M, Ma G, Deng Y, Zhang J, et al. NSUN2 lactylation drives cancer cell resistance to ferroptosis through enhancing GCLC-dependent glutathione synthesis. Redox Biol. 2025;79:103479. 39742570 10.1016/j.redox.2024.103479 PMC11750563

[46] Chen Y, Yan Q, Ruan S, Cui J, Li Z, Zhang Z, et al. GCLM lactylation mediated by ACAT2 promotes ferroptosis resistance in KRAS(G12D)-mutant cancer. Cell Rep. 2025;44:115774. 40503938 10.1016/j.celrep.2025.115774

[47] Yang T, Zhang S, Nie K, Cheng C, Peng X, Huo J, et al. ZNF207-driven PRDX1 lactylation and NRF2 activation in regorafenib resistance and ferroptosis evasion. Drug Resist Updat. 2025;82:101274. 40680452 10.1016/j.drup.2025.101274

[48] Lin J, Yin Y, Cao J, Zhang Y, Chen J, Chen R, et al. NUDT21 lactylation reprograms alternative polyadenylation to promote cuproptosis resistance. Cell Discov. 2025;11:52. 40425546 10.1038/s41421-025-00804-1 PMC12116747

[49] Sun L, Zhang Y, Yang B, Sun S, Zhang P, Luo Z, et al. Lactylation of METTL16 promotes cuproptosis via m(6)A-modification on FDX1 mRNA in gastric cancer. Nat Commun. 2023;14:6523. 37863889 10.1038/s41467-023-42025-8 PMC10589265

[50] Li W, Zhou C, Yu L, Hou Z, Liu H, Kong L, et al. Tumor-derived lactate promotes resistance to bevacizumab treatment by facilitating autophagy enhancer protein RUBCNL expression through histone H3 lysine 18 lactylation (H3K18la) in colorectal cancer. Autophagy. 2024;20:114–30. 37615625 10.1080/15548627.2023.2249762 PMC10761097

[51] Jia M, Yue X, Sun W, Zhou Q, Chang C, Gong W, et al. ULK1-mediated metabolic reprogramming regulates Vps34 lipid kinase activity by its lactylation. Sci Adv. 2023;9:eadg4993. 37267363 10.1126/sciadv.adg4993 PMC10413652

[52] Sun W, Jia M, Feng Y, Cheng X. Lactate is a bridge linking glycolysis and autophagy through lactylation. Autophagy. 2023;19:3240–1. 37565742 10.1080/15548627.2023.2246356 PMC10621282

[53] Feng F, Wu J, Chi Q, Wang S, Liu W, Yang L, et al. Lactylome analysis unveils lactylation-dependent mechanisms of stemness remodeling in the liver cancer stem cells. Adv Sci. 2024;11:e2405975. 10.1002/advs.202405975 PMC11481176 39099416

[54] Yan F, Teng Y, Li X, Zhong Y, Li C, Yan F, et al. Hypoxia promotes non-small cell lung cancer cell stemness, migration, and invasion via promoting glycolysis by lactylation of SOX9. Cancer Biol Ther. 2024;25:2304161. 38226837 10.1080/15384047.2024.2304161 PMC10793688

[55] Zhou Z, Yin X, Sun H, Lu J, Li Y, Fan Y, et al. PTBP1 lactylation promotes glioma stem cell maintenance through PFKFB4-driven glycolysis. Cancer Res. 2025;85:739–57. 39570804 10.1158/0008-5472.CAN-24-1412

[56] He J, Li W, Wang S, Lan J, Hong X, Liao L, et al. Cancer associated fibroblasts-derived lactate induces oxaliplatin treatment resistance by promoting cancer stemness via ANTXR1 lactylation in colorectal cancer. Cancer Lett. 2025;631:217917. 40683418 10.1016/j.canlet.2025.217917

[57] Liu Y, Chen J, Ma L, Zhao S, Hui X, Xiong W, et al. ZMIZ1 lactylation induces tamoxifen resistance in breast cancer through increasing transcriptional activity of Nanog to impact cell stemness and cholesterol uptake. Cell Biol Toxicol. 2025;41:117. 40670831 10.1007/s10565-025-10068-w PMC12267387

[58] Dhimolea E, de Matos Simoes R, Kansara D, Al’Khafaji A, Bouyssou J, Weng X, et al. An embryonic diapause-like adaptation with suppressed Myc activity enables tumor treatment persistence. Cancer Cell. 2021;39:240–56 e11. 33417832 10.1016/j.ccell.2020.12.002 PMC8670073

[59] Sun X, He L, Liu H, Thorne RF, Zeng T, Liu L, et al. The diapause-like colorectal cancer cells induced by SMC4 attenuation are characterized by low proliferation and chemotherapy insensitivity. Cell Metab. 2023;35:1563–79 e8. 37543034 10.1016/j.cmet.2023.07.005

[60] Huang ZW, Zhang XN, Zhang L, Liu LL, Zhang JW, Sun YX, et al. STAT5 promotes PD-L1 expression by facilitating histone lactylation to drive immunosuppression in acute myeloid leukemia. Signal Transduct Target Ther. 2023;8:391. 37777506 10.1038/s41392-023-01605-2 PMC10542808

[61] Zhang C, Zhou L, Zhang M, Du Y, Li C, Ren H, et al. H3K18 lactylation potentiates immune escape of non-small cell lung cancer. Cancer Res. 2024;84:3589–601. 39137401 10.1158/0008-5472.CAN-23-3513

[62] Ding CH, Yan FZ, Xu BN, Qian H, Hong XL, Liu SQ, et al. PRMT3 drives PD-L1-mediated immune escape through activating PDHK1-regulated glycolysis in hepatocellular carcinoma. Cell Death Dis. 2025;16:158. 40050608 10.1038/s41419-025-07482-7 PMC11885674

[63] Tong H, Jiang Z, Song L, Tan K, Yin X, He C, et al. Dual impacts of serine/glycine-free diet in enhancing antitumor immunity and promoting evasion via PD-L1 lactylation. Cell Metab. 2024;36:2493–510.e9. 39577415 10.1016/j.cmet.2024.10.019

[64] Li XM, Yang Y, Jiang FQ, Hu G, Wan S, Yan WY, et al. Histone lactylation inhibits RARgamma expression in macrophages to promote colorectal tumorigenesis through activation of TRAF6-IL-6-STAT3 signaling. Cell Rep. 2024;43:113688. 38245869 10.1016/j.celrep.2024.113688

[65] Cai J, Song L, Zhang F, Wu S, Zhu G, Zhang P, et al. Targeting SRSF10 might inhibit M2 macrophage polarization and potentiate anti-PD-1 therapy in hepatocellular carcinoma. Cancer Commun. 2024;44:1231–60. 10.1002/cac2.12607 PMC11570766 39223929

[66] Xiong J, He J, Zhu J, Pan J, Liao W, Ye H, et al. Lactylation-driven METTL3-mediated RNA m(6)A modification promotes immunosuppression of tumor-infiltrating myeloid cells. Mol Cell. 2022;82:1660–77 e10. 35320754 10.1016/j.molcel.2022.02.033

[67] Dang T, You Y, Wei L, Li Q, Sun H, Sun M, et al. ICAT drives lactylation of tumor-associated macrophages via the c-Myc-ENO1 axis to promote cervical cancer progression. Free Radic Biol Med. 2025;241:316–29. 40976412 10.1016/j.freeradbiomed.2025.09.031

[68] Yang J, Yu X, Xiao M, Xu H, Tan Z, Lei Y, et al. Histone lactylation-driven feedback loop modulates cholesterol-linked immunosuppression in pancreatic cancer. Gut. 2025;74:1859–72. 40467104 10.1136/gutjnl-2024-334361 PMC12573332

[69] Sun T, Liu B, Li Y, Wu J, Cao Y, Yang S, et al. Oxamate enhances the efficacy of CAR-T therapy against glioblastoma via suppressing ectonucleotidases and CCR8 lactylation. J Exp Clin Cancer Res. 2023;42:253. 37770937 10.1186/s13046-023-02815-w PMC10540361

[70] Xue Q, Peng W, Zhang S, Wei X, Ye L, Wang Z, et al. Lactylation-driven TNFR2 expression in regulatory T cells promotes the progression of malignant pleural effusion. J Immunother Cancer. 2024;12:e010040. 39721754 10.1136/jitc-2024-010040 PMC11683941

[71] Gu J, Zhou J, Chen Q, Xu X, Gao J, Li X, et al. Tumor metabolite lactate promotes tumorigenesis by modulating MOESIN lactylation and enhancing TGF-beta signaling in regulatory T cells. Cell Rep. 2022;39:110986. 35732125 10.1016/j.celrep.2022.110986

[72] Chen J, Zhao D, Wang Y, Liu M, Zhang Y, Feng T, et al. Lactylated apolipoprotein C-II induces immunotherapy resistance by promoting extracellular lipolysis. Adv Sci. 2024;11:e2406333. 10.1002/advs.202406333 PMC11481198 38981044

[73] Ugolini A, De Leo A, Yu X, Scirocchi F, Liu X, Peixoto B, et al. Functional reprogramming of neutrophils within the brain tumor microenvironment by hypoxia-driven histone lactylation. Cancer Discov. 2025;15:1270–96. 40014923 10.1158/2159-8290.CD-24-1056 PMC12133432

[74] Wang R, Li C, Cheng Z, Li M, Shi J, Zhang Z, et al. H3K9 lactylation in malignant cells facilitates CD8(+) T cell dysfunction and poor immunotherapy response. Cell Rep. 2024;43:114686. 39216002 10.1016/j.celrep.2024.114686

[75] Zhou C, Li W, Liang Z, Wu X, Cheng S, Peng J, et al. Mutant KRAS-activated circATXN7 fosters tumor immunoescape by sensitizing tumor-specific T cells to activation-induced cell death. Nat Commun. 2024;15:499. 38216551 10.1038/s41467-024-44779-1 PMC10786880

[76] Ma Z, Yang J, Jia W, Li L, Li Y, Hu J, et al. Histone lactylation-driven B7-H3 expression promotes tumor immune evasion. Theranostics. 2025;15:2338–59. 39990209 10.7150/thno.105947 PMC11840737

[77] Jin J, Yan P, Wang D, Bai L, Liang H, Zhu X, et al. Targeting lactylation reinforces NK cell cytotoxicity within the tumor microenvironment. Nat Immunol. 2025;26:1099–112. 40494934 10.1038/s41590-025-02178-8

[78] Luo Y, Yang Z, Yu Y, Zhang P. HIF1alpha lactylation enhances KIAA1199 transcription to promote angiogenesis and vasculogenic mimicry in prostate cancer. Int J Biol Macromol. 2022;222:2225–43. 36209908 10.1016/j.ijbiomac.2022.10.014

[79] Han H, Wang S, Ma L, Yin H, Cheng X, Wang Y. ASH2L-K312-Lac stimulates angiogenesis in tumors to expedite the malignant progression of hepatocellular carcinoma. Adv Sci. 2025;12:e09477. 10.1002/advs.202509477 PMC12561209 40726441

[80] Zhao M, Qian Y, He L, Peng T, Wang H, Wang X, et al. Lactate-mediated histone lactylation promotes melanoma angiogenesis via IL-33/ST2 axis. Cell Death Dis. 2025;16:701. 41053006 10.1038/s41419-025-08023-y PMC12501017

[81] Sun K, Zhang X, Shi J, Huang J, Wang S, Li X, et al. Elevated protein lactylation promotes immunosuppressive microenvironment and therapeutic resistance in pancreatic ductal adenocarcinoma. J Clin Invest. 2025;135:e187024. 39883522 10.1172/JCI187024 PMC11957693

[82] Cheung SM, Husain E, Masannat Y, Miller ID, Wahle K, Heys SD, et al. Lactate concentration in breast cancer using advanced magnetic resonance spectroscopy. Br J Cancer. 2020;123:261–7. 32424149 10.1038/s41416-020-0886-7 PMC7374160

[83] Dutta P, Perez MR, Lee J, Kang Y, Pratt M, Salzillo TC, et al. Combining hyperpolarized real-time metabolic imaging and NMR spectroscopy to identify metabolic biomarkers in pancreatic cancer. J Proteome Res. 2019;18:2826–34. 31120258 10.1021/acs.jproteome.9b00132

[84] Zhu L, Ruan J, Zhang Q, Feng L, Deng Y, Dai L, et al. LDH and glycolytic activity as predictors of immunotherapy response in gastric cancer: a systematic review and meta-analysis. Front Immunol. 2025;16:1605976. 40552288 10.3389/fimmu.2025.1605976 PMC12183248

[85] Passardi A, Scarpi E, Tamberi S, Cavanna L, Tassinari D, Fontana A, et al. Impact of pre-treatment lactate dehydrogenase levels on prognosis and bevacizumab efficacy in patients with metastatic colorectal cancer. PLoS One. 2015;10:e0134732. 26244985 10.1371/journal.pone.0134732 PMC4526665

[86] Keilholz U, Martus P, Punt CJ, Kruit W, Mooser G, Schadendorf D, et al. Prognostic factors for survival and factors associated with long-term remission in patients with advanced melanoma receiving cytokine-based treatments: second analysis of a randomised EORTC Melanoma Group trial comparing interferon-alpha2a (IFNalpha) and interleukin 2 (IL-2) with or without cisplatin. Eur J Cancer. 2002;38:1501–11. 12110497 10.1016/s0959-8049(02)00123-5

[87] Uysal M, Bozcuk H, Sezgin Göksu S, Murat Tatli A, Arslan D, Gündüz S, et al. Basal proteinuria as a prognostic factor in patients with metastatic colorectal cancer treated with bevacizumab. Biomed Pharmacother. 2014;68:409–12. 24721326 10.1016/j.biopha.2014.03.002

[88] Witzig TE, Vukov AM, Habermann TM, Geyer S, Kurtin PJ, Friedenberg WR, et al. Rituximab therapy for patients with newly diagnosed, advanced-stage, follicular grade I non-Hodgkin’s lymphoma: a phase II trial in the North Central Cancer Treatment Group. J Clin Oncol. 2005;23:1103–8. 15657404 10.1200/JCO.2005.12.052

[89] Gaffney DO, Jennings EQ, Anderson CC, Marentette JO, Shi T, Schou Oxvig AM, et al. Non-enzymatic lysine lactoylation of glycolytic enzymes. Cell Chem Biol. 2020;27:206–13 e6. 31767537 10.1016/j.chembiol.2019.11.005 PMC7395678

[90] Trujillo MN, Jennings EQ, Hoffman EA, Zhang H, Phoebe AM, Mastin GE, et al. Lactoylglutathione promotes inflammatory signaling in macrophages through histone lactoylation. Mol Metab. 2024;81:101888. 38307385 10.1016/j.molmet.2024.101888 PMC10869261

[91] Yang Z, Su W, Zhang Q, Niu L, Feng B, Zhang Y. Lactylation of HDAC1 confers resistance to ferroptosis in colorectal cancer. Adv Sci. 2025;12:e2408845. 10.1002/advs.202408845 PMC11947995 39888307

[92] Duan W, Liu W, Xia S, Zhou Y, Tang M, Xu M, et al. Warburg effect enhanced by AKR1B10 promotes acquired resistance to pemetrexed in lung cancer-derived brain metastasis. J Transl Med. 2023;21:547. 37587486 10.1186/s12967-023-04403-0 PMC10428599

[93] Cheng S, Chen L, Ying J, Wang Y, Jiang W, Zhang Q, et al. 20(S)-ginsenoside Rh2 ameliorates ATRA resistance in APL by modulating lactylation-driven METTL3. J Ginseng Res. 2024;48:298–309. 38707638 10.1016/j.jgr.2023.12.003 PMC11068957

[94] Lu Y, Zhu J, Zhang Y, Li W, Xiong Y, Fan Y, et al. Lactylation-driven IGF2BP3-mediated serine metabolism reprogramming and RNA m6A-modification promotes lenvatinib resistance in HCC. Adv Sci. 2024;11:e2401399. 10.1002/advs.202401399 PMC11633555 39450426

